# Hydroxyurea Improves Spatial Memory and Cognitive Plasticity in Mice and Has a Mild Effect on These Parameters in a Down Syndrome Mouse Model

**DOI:** 10.3389/fnagi.2019.00096

**Published:** 2019-05-14

**Authors:** Rebecca Deering Brose, Alena Savonenko, Benjamin Devenney, Kirby D. Smith, Roger H. Reeves

**Affiliations:** ^1^Department of Physiology, Johns Hopkins University School of Medicine, Baltimore, MD, United States; ^2^Departments of Pathology and Neurology, Johns Hopkins University School of Medicine, Baltimore, MD, United States; ^3^McKusick-Nathans Institute of Genetic Medicine, Baltimore, MD, United States

**Keywords:** Down syndrome, trisomy, hydroxyurea, adaptive stress response, neurodegeneration, nootropic effect, reference memory, episodic-like memory

## Abstract

Down syndrome (DS), a genetic disorder caused by partial or complete triplication of chromosome 21, is the most common genetic cause of intellectual disability. DS mouse models and cell lines display defects in cellular adaptive stress responses including autophagy, unfolded protein response, and mitochondrial bioenergetics. We tested the ability of hydroxyurea (HU), an FDA-approved pharmacological agent that activates adaptive cellular stress response pathways, to improve the cognitive function of Ts65Dn mice. The chronic HU treatment started at a stage when early mild cognitive deficits are present in this model (∼3 months of age) and continued until a stage of advanced cognitive deficits in untreated mice (∼5–6 months of age). The HU effects on cognitive performance were analyzed using a battery of water maze tasks designed to detect changes in different types of memory with sensitivity wide enough to detect deficits as well as improvements in spatial memory. The most common characteristic of cognitive deficits observed in trisomic mice at 5–6 months of age was their inability to rapidly acquire new information for long-term storage, a feature akin to episodic-like memory. On the background of severe cognitive impairments in untreated trisomic mice, HU-treatment produced mild but significant benefits in Ts65Dn by improving memory acquisition and short-term retention of spatial information. In control mice, HU treatment facilitated memory retention in constant (reference memory) as well as time-variant conditions (episodic-like memory) implicating a robust nootropic effect. This was the first proof-of-concept study of HU treatment in a DS model, and indicates that further studies are warranted to assess a window to optimize timing and dosage of the treatment in this pre-clinical phase. Findings of this study indicate that HU has potential for improving memory retention and cognitive flexibility that can be harnessed for the amelioration of cognitive deficits in normal aging and in cognitive decline (dementia) related to DS and other neurodegenerative diseases.

## Introduction

Individuals with Down syndrome (DS) have a partial or complete extra copy of human chromosome 21 (trisomy 21; HSA21). DS is the most common aneuploidy compatible with survival, occurring in 1 out of 691 live births (Centers for Disease Control and Prevention [CDC], 2017). People with DS commonly display intellectual disability, hypotonia, and delayed language and speech development (Mundy et al., 1995; Pavarino Bertelli et al., 2009). The DS brain is characterized by a small cerebellum, reduced neurogenesis, dendritic hypotrophy, and altered neurotransmitter and receptor systems (Das et al., 2013; Stagni et al., 2015). Degeneration of basal forebrain cholinergic neurons, increased microglial activation, cognitive decline, and Alzheimer’s disease neuropathology and dementia develop with age (Bimonte-Nelson et al., 2003).

One of a multitude of DS-associated deficiencies that recently attracted more attention is the dysregulation of the adaptive cellular stress response. It involves several interconnected signaling pathways, including mitochondrial bioenergetics, autophagy, the antioxidant response, and the unfolded protein response. Trisomic cells from DS or DS mouse models exhibit mitochondrial dysfunction, increased oxidative stress and damage, and mTOR pathway hyperactivation leading to reduced autophagy (Busciglio et al., 2002; Helguera et al., 2013; Cenini et al., 2014; Perluigi et al., 2014; Tramutola et al., 2015, 2016; Liu et al., 2017). Many disomic genes are dysregulated as a result of trisomy, and these are enriched for genes related to oxidative stress and mitochondrial function, including *NFE2L2*-associated genes (Helguera et al., 2013). Further, brain tissue from individuals with DS exhibits reduced autophagosome formation, reducing the ability of the brain to clear damaged proteins and organelles (Di Domenico et al., 2013). Importantly, these effects on the proteostasis network are found in individuals with DS years before age-related cognitive decline and Alzheimer’s-like dementia are detected. This represents a large pharmaceutical treatment window to delay and/or improve the long-term cognitive function of individuals with DS. There is no available treatment to improve intellectual disabilities or age-related dementia in individuals with DS. Improvement in the pathways of adaptive stress response may present a novel therapeutic opportunity and is particularly attractive as treatments targeting these pathways have already been approved for non-DS related clinical use.

Hydroxyurea (HU), an FDA-approved ribonucleotide reductase inhibitor, is known to improve cellular homeostasis through stimulation of mitochondrial bioenergetics, autophagy, and the antioxidant response (Brose et al., 2012). In our previous work we showed that *in vitro*, HU protects primary rat hippocampal neurons against increased oxidative stress, mitochondrial stress, and excitotoxicity (Brose et al., 2018). *In vivo*, HU treatment of *APPswe/PS1dE9* mice, an Alzheimer’s disease model, ameliorated deficits in spatial memory tested in a hippocampus-dependent Morris water maze (MWM) task (Brose et al., 2018). We hypothesized that HU treatment would improve cognitive deficits in a mouse model of DS, as well. To test our hypothesis, we chose the Ts65Dn mouse model which is trisomic for the distal portion of mouse chromosome 16 (MMU16) containing approximately 94 genes orthologous to HSA21. Ts65Dn mice have brain dysmorphology, transcriptional and biochemical changes as well as cognitive deficits that mirror several anomalies observed in individuals with DS (Davisson et al., 1993; Reeves et al., 1995; Kahlem et al., 2004). This is the most widely used DS mouse model to date for the preclinical study of therapeutic treatments for DS.

The Ts65Dn model has been used extensively in different behavioral tests, including the Y-maze, novel object recognition test, MWM, and fear conditioning (Das et al., 2013; Dutka et al., 2015; Olmos-Serrano et al., 2016). Consistent among different research groups, the Ts65Dn mice have severely impaired spatial learning and memory, measurable by their inability to learn and remember the location of the hidden platform in the MWM. This deficit is correlated with significant impairment of long term potentiation in the dentate gyrus of the hippocampus (Siarey et al., 1997; Belichenko et al., 2004; Ruparelia et al., 2012). Behavioral tests with consistently reproducible DS-related phenotypes, such as the MWM, have been used to evaluate the ability of pharmacological interventions to improve cognitive measures in DS mouse models (Reeves et al., 1995; Moran et al., 2002; Stasko and Costa, 2004; Das and Reeves, 2011; Velazquez et al., 2013). The usual caveats of using an animal model in the preclinical stage of testing must, of course, be applied to the Ts65Dn model. From a genetic perspective, it has been known since the model was created that it is trisomic for only about 60% of mouse orthologs of Hsa21 genes. More recently, whole genome sequencing has documented the presence of a number of trisomic genes whose orthologs are on human chromosomes other than 21 (Duchon et al., 2011; Reinholdt et al., 2011).

To test our hypothesis, we treated Ts65Dn mice with HU and monitored cognitive performance using a battery of water maze tasks originally designed to detect changes in different types of memory in normal aging and in aging aggravated by Aβ amyloidosis in AD mouse models (Jankowsky et al., 2005; Savonenko et al., 2005). These tasks have been used successfully to test several different experimental treatments (Laird et al., 2005; Savonenko et al., 2009; Chow et al., 2010; Tabatadze et al., 2010) with sensitivity wide enough to detect deficits as well as improvements in spatial memory. Our experiments demonstrated that chronic HU treatment resulted in mild but significant improvements of cognitive deficits in the Ts65Dn mice, while in wild type control mice the treatment had clear nootropic effects, significantly facilitating learning and memory.

## Materials and Methods

### Study Design

**FIGURE 1:**
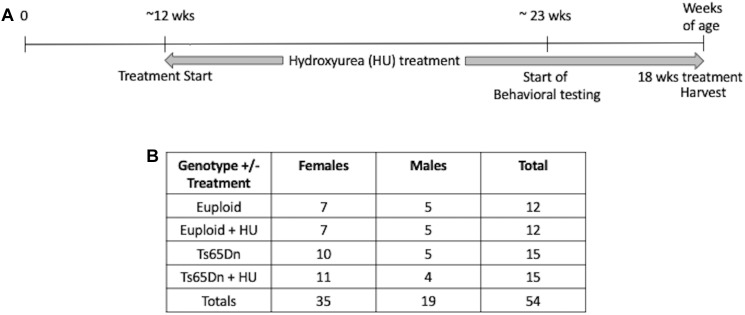
Study design. **(A)** A proof-of-concept study to monitor the preventative effects of hydroxyurea (HU) on cognitive function in Ts65Dn mice. HU (30 mg/kg/day dissolved in water) treatment started at ∼12 weeks of age and continued for at least 10 weeks before behavioral testing began. Mice were harvested after 18 weeks of HU treatment. **(B)** Number of cases per genotype per treatment. During the study two untreated male euploid mice and one untreated Ts65Dn male died.

This study was carried out in accordance with the recommendations of the NIH Guide for the Care and Use of Laboratory Animals and the Johns Hopkins University Institute of Animal Care and Use Committee. The protocol was approved by the Johns Hopkins University Institute of Animal Care and Use Committee. Ts65Dn mice were maintained by our laboratory as an advanced intercross on a C57BL/6J x C3H/HeJ F_n_ background. No mice homozygous for the *Pde6b(rd)1* retinal degeneration mutant allele were used in this study. Investigators performing the testing were blind to genotype. The study design is shown in Figure 1. This study began with 54 mice: 15 Ts65Dn mice (10 females, 5 males), 15 Ts65Dn mice treated with HU (11 females, 4 males), 12 euploid littermates (7 females, 5 males), 12 euploid littermates treated with HU (7 females, 5 males). Three untreated mice died during this study, two male euploid mice and one male Ts65Dn mouse.

### Drug Treatment and Dose Selection

The dosage of HU was based on previous studies focused on the effects of hydroxyurea in different mouse models. Sickle cell disease mouse models were given 50 mg/kg/day of HU to induce fetal hemoglobin and reduce leukocyte–endothelial interactions (Lebensburger et al., 2012). *APPswe/PS1dE9* mice were treated with 45 mg/kg/day to improve spatial memory in the MWM (Brose et al., 2018). Hydroxyurea is an FDA-approved drug for several diseases. Humans with sickle cell disease, HIV, or psoriasis are treated with 15 – 35 mg/kg/day (Medscape, 2018). Based on this information, we used a conservative dose of 30 mg/kg/day of HU in their drinking water for 18 weeks. The HU was refreshed weekly. Control groups received water alone (untreated) for 18 weeks. Water intake per cage was monitored weekly. HU crosses the blood-brain barrier; its brain uptake clearance is 0.10 microl/g/s in mice (Dogruel et al., 2003; Bihorel et al., 2006; Syvanen et al., 2007).

This is the first proof-of-concept study of hydroxyurea treatment in the Ts65Dn model. The treatment started at a stage when only early mild cognitive deficits are present (Olmos-Serrano et al., 2016). In this study, the mice were approximately 3 months of age (mean ± SEM = 11.8 ± 0.3 weeks, range 7–14 weeks) when HU treatment began. Mouse handling and behavioral testing began after at least 10 weeks of HU treatment (range 10–13 weeks). Treatment was continued throughout behavioral testing. After 18 weeks of HU treatment, the mice were euthanized and their organs harvested.

### Behavioral Testing

Behavioral testing started when mice reached approximately 6 months of age (23.3 ± 1.8 weeks; see Supplementary Figure S1). Mice were handled for several minutes a day for four consecutive days at least 1 week prior to the start of behavioral testing to familiarize them with handling. Two HU-treated Ts65Dn mice were excluded from behavioral testing due to cataracts (one male, one female) and one female HU-treated Ts65Dn mouse was excluded from the RRWM and RAWM because of low body weight (less than 19 g). Tests were performed in the order described below. ANY-maze 5.1 behavioral tracking software (Stoelting Co.; Wood Dale, IL, United States) was used for video-recording and executing each test.

### Water Maze Tasks

Water maze tasks were performed as described before (Jankowsky et al., 2005; Savonenko et al., 2005) with small modifications. For all water maze tasks, the water was 20–22^°^C and made opaque by adding non-toxic white tempera paint to hide a rectangular platform (10 cm × 10 cm) 2 cm below the water surface. The mice were tested in groups of 8–10 mice with an inter-trial interval of 20 min. Each trial was performed for all mice in a group before starting the next trial. If the platform was not found, the mouse was guided visually by placing a finger on top of the platform or by manually guiding the mouse to the platform. Mice were dried between trials and returned to a dry waiting cage.

#### Platform Pretraining

Mice were first taught to find a platform as an escape from a small pool of opaque water (55 cm diameter) over five trials. For the first trial, each mouse was placed directly next to the platform. For trial 2, each mouse was placed one to two inches away from the platform. For trials 3–5, each mouse was placed halfway between the platform and the wall of the pool.

#### Straight Swim Pretraining

A platform hidden 2 cm below opaque water was placed at the end of a straight alley (15 × 110 cm). For five trials, each mouse was placed in the end of the alley opposite the platform and given 60 s to find the platform. If the platform was not found, the mouse was guided visually as above. The purpose of this and the previous task was to check for possible deficits in swimming abilities as well as to train the mice to find and stay on a hidden platform as preparation for the subsequent tasks.

#### Classic Morris Water Maze (MWM)

**FIGURE 2:**
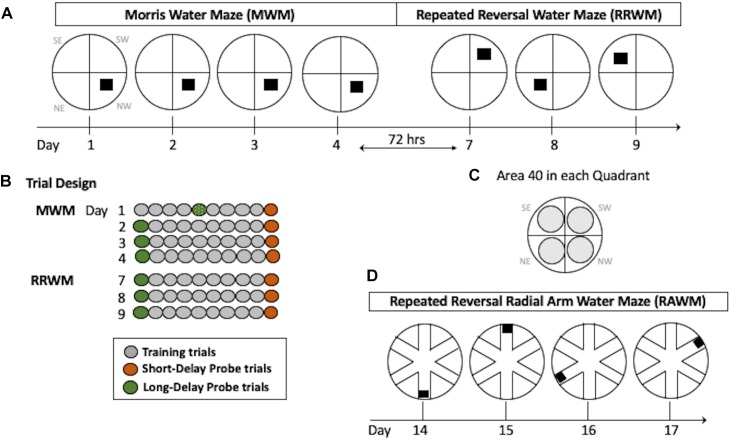
Water maze paradigms. **(A)** The MWM task was performed for four consecutive days with the hidden platform in the same location. Seventy-two hours after Day 4, the RRWM task was started in which the location of the hidden platform was changed each day for three days as depicted. **(B)** Daily trial design. Each day, mice had 8 training trials. In addition, two daily probe trials (at the beginning and the end of the day) were introduced in which the platform was lowered for a variable interval (30–45 s). Since mice were naive, on day 1 the first probe trial of the day was performed after four training trials. **(C)** Areas 40 cm in diameter (gray circles) are shown for each quadrant of the water maze. These areas were used to calculate spatial preferences in the MWM and the RRWM probe trials (see Stat Analyses section for more details). Day designations 1–17 correlate to chronological experimental days. Quadrant designations in **(A,C)** panels: NE, northeast; NW, northwest; SW, southwest; and SE, southeast. **(D)** The RAWM task was performed for four consecutive days consisting of 6 trials. The location of the hidden platform was changed daily.

This task requires incremental learning of a constant platform location over multiple days of training using the same set of spatial cues. Learning in this task results in formation of long-lasting reference memory (Morris, 2001; Nonaka et al., 2017). The duration of the task was 4 days with the order of the trials as shown in Figure 2. For each trial, the mouse was placed in a 110 cm pool facing the wall in a randomly predetermined quadrant other than the quadrant containing the platform. Inter-trial intervals were approximately 20 min. Training trials were 60 s long with the hidden platform in its upright position two centimeters below the water surface and available for a mouse to climb on. If a mouse did not find the platform during a training trial it was either visually or manually guided to the platform. Probe trials were conducted with the platform in its lowered position and unavailable for climbing for a variable interval (30–45 s). At the end of this interval, the collapsed platform was returned to its raised position to maintain the same response-reinforcement contingency as in the platform trials, allowing the use of probe trials repeatedly without the effect of extinction (Markowska et al., 1993; Andreasson et al., 2001; Micheau et al., 2004). The probe trials were conducted at the beginning and the end of a daily session, and therefore they assessed memory following short (30 min) and long delays (24 h, the first trial of each daily session except Day 1; Figure 2). The last probe trial for this task was run after a 72-h delay and this trial initiated the testing in the RRWM task (see below and Figure 2). This design of the probe trials increases sensitivity of the task by detecting deficits or improvements in short- vs. long-term spatial memory as well as accessing memory acquisition at different stages of spatial learning (Andreasson et al., 2001).

#### Repeated Reversal Water Maze (RRWM)

In contrast to the reference memory task (MWM), where mice are required to remember the same platform location over several days of training, the RRWM task challenged episodic-like memory as mice need to keep changing their memory representation for the environment by discriminating the information by “what,” “where” and “when” categories (Morris, 2001; Nonaka et al., 2017). This task was performed similarly to the MWM except the platform location was changed daily for three testing days (Figure 2). Each day consisted of a probe trial, followed by eight training trials, and a final probe trial. The start location for each trial was randomly predetermined and the probe trials were always started in a location that the platform had not been in the previous testing day.

#### Radial Arm Water Maze (RAWM)

After completion of the RRWM task, a radial maze enclosure was placed into the same pool and the mice were required to find the hidden platform in one of the six arms of the radial water maze (Figure 2D). Similar to the repeated reversals, the position of the platform was changed daily. Originally sought as only a working memory task, RAWM also includes episodic-like features and, similar to the repeated reversals task, requires use of “where” and “when” categories in an integrated and flexible manner (Savonenko et al., 2005). Procedural aspects of the RAWM task were similar to the previous ones since mice used the same set of spatial cues as in the previous water maze tasks and learned a new platform location every day as in the repeated reversals task (Figure 2). For a total of 4 days, each mouse performed six 120 s trials; each trial was started in a randomly predetermined arm. In addition to recording by the AnyMaze software, the tester documented the arms the mouse entered before finding the platform during each trial. Errors were counted as entries into the arms that do not contain the platform or as entries to a correct arm (containing the platform) with a failure to find the platform.

### Statistical Analyses

The data were analyzed using the statistical package STATISTICA 13 (TIBCO Software Inc, Palo Alto, CA, United States) and a minimal level of significance *p* < 0.05. Based on our previous studies using the three-tier battery of water maze tasks, the primary outcomes were chosen to ensure the sensitivity of the tests without inflating the number of comparisons. The outcome measures were latency and distance to find the platform, swim speed (all tasks), percent of time spent in Areas 40 (MWM and RRWM), and number of errors (RAWM). The Areas 40 were the areas with a 40 cm diameter centered on the platform locations used in the MWM and RRWM tasks (Figure 2C). The sum of all Areas 40 represented only 67% of the pool area that allowed statistical analyses of all four areas without violating degrees of freedom. Therefore, the chance level of swimming in any one of the four Areas 40 was 16%. The data were initially analyzed using three-way repeated measures analyses of variance (RM-ANOVA) with Sex and Group (EU, EU+HU, TS, TS+HU) as independent factors and a repeated measure, RM (trials, blocks, arms, etc.). Sex × Group, Group × RM, and Sex × RM interactions were set as orthogonal.

Only one outcome measure, the latency to find the platform, was significantly modified by Sex because male mice swam with shorter latencies/higher swimming speed. To avoid this interference, we report distance traveled to the platform instead of latency. Importantly, no other than latency outcome measures yielded significant effects of Sex or interactions of Sex with Group or RM. Based on these findings, the data were reported based on results of two-way RM-ANOVAs with Group as a single main factor. The LSD *post hoc* test was applied to significant Group or Group × RM interactions to evaluate differences between four sets of means: EU vs. EU+HU; EU vs. TS; EU vs. TS+HU; TS vs. TS+HU. Two-tailed *t*-test was used to analyze differences from chance levels. The summary of statistical analyses is presented in Table 1. The details of statistical results including *F*, df, and *post hoc* tests are presented in Supplementary Tables S1–S3. Data in figures represent means ± standard error of means (SEM) unless otherwise noted.

**Table 1:** Summary of statistical analyses for the Morris Water Maze (MWM), repeated reversal water maze (RRWM), and the repeated reversal radial arm water maze (RR-RAWM).

Figure	Task	Measurement	RM-ANOVA	Fisher’s LSD *post hoc*
3A	MWM	Distance to platform by trial Day 1	*p*_trial_ < 1 × e^−6^	EU or EU + HU vs. TS or TS + HU *p* < 0.004
3B	MWM	Distance to platform across days	*p* < 1 × e^−6^	EU or EU + HU vs. TS or TS + HU *p* < 1 × e^−6^
3C	MWM	Percent time in NW Area 40 during 30-min short-delay probe trial	*p* < 1 × e^−6^	EU or EU + HU vs. TS or TS + HU *p* < 0.022
3C	MWM	Percent time in NW Area 40 during 30-min short-delay probe trial day 1 vs. day 4	*p* = 0.006	*p*_EU,EU__+__HU,TS__+__HU_ ≤ 0.016 *p*_TS_ = 0.238
3D	MWM	Percent time in NW Area 40 during 30-min short-delay probe trial, day 4	*p* = 6 × e^−6^	EU or EU + HU vs. TS or TS + HU *p* ≤ 2 × e^−5^
3D	MWM	Percent time in NW Area 40 during 30-min short-delay probe trial on day 4 compared to chance level	*p* = 6 × e^−6^	NW_EU_, NW_EU__+__HU_, or NW_TS__+__HU_ *p* ≤ 0.022 and NW_TS__+__HU_ *p* = 0.180
3E	MWM	Percent time in NW Area 40 during 24-h long-delay probe trial	*p* = 6 × e^−6^	EU vs. TS or TS + HU *p* ≤ 2 × e^−4^
3F	MWM	Percent time in NW Area 40 after 72-h delay	*p* = 0.010	EU + HU vs. EU, TS, or TS + HU *p* ≤ 3.9 × e^−4^
3F	MWM	Percent time in NW Area 40 after 72-h delay compared to other quadrant areas	*p* = 1.5 × e^−3^	NW_EU__+__HU_ vs. NE, NW, or SW *p* ≤ 1.2 × e^−5^
4A	RRWM	Distance to platform per trial	*p* < 1 × e^−6^	EU or EU + HU vs. TS or TS + HU *p* ≤ 0.004; TS + HU vs. TS *p* = 0.044
4B	RRWM	Average distance trials 4–8	*p* < 1 × e^−6^	TS + HU vs. TS *p* = 0.108TS or TS + HU *p* ≤ 0.108
4C	RRWM	Percent time in Area 40 platform areas during 30-min short-delay probe trials	*p* < 1 × e^−6^	EU or EU + HU vs. TS or TS + HU *p* ≤ 5.6 × e^−4^
4D	RRWM	Percent time in the previous day’s Area 40 platform area, 24-h long-delay probe trials	*p* < 1 × e^−6^	EU or EU + HU vs. TS or TS + HU *p* ≤ 0.011; EU + HU vs. EU *p* ≤ 4.2 × e^−4^
5A	RR-RAWM	Distance to platform per trial	*p*_group_ = 0.388 *p*_trial_ < 1 × e^−6^	
5B	RR-RAWM	Average distance trials 4–6	*p* = 0.016	EU vs. TS *p* = 0.018; TS + HU vs. TS *p* = 0.115
5C	RR-RAWM	Average arm entry errors per trial	*p*_trial∗group_ < 1 × e^−6^	
5D	RR-RAWM	Average arm entry errors trials 4–6	*p* < 1.3 × e^−3^	EU, EU + HU, or TS + HU vs. TS *p* < 0.03
5E	RR-RAWM	Errors due to swimming in previous day’s platform location, trial 1	*p* = 3.1 × e^−3^	EU or EU + HU vs. TS or TS + HU *p* ≤ 0.040
5F	RR-RAWM	Average previous platform errors trial 1	*p* = 3.2 × e^−3^	EU or EU + HU vs. TS or TS + HU *p* ≤ 0.040

*EU, euploid; EU + HU, HU-treated euploid; TS, Ts65Dn; TS + HU, HU-treated Ts65Dn; NW, northwest. See detailed statistical results in Supplementary Tables S1–S3.*

## Results

To determine the cognitive differences between Ts65Dn mice and their euploid littermates and to more sensitively detect possible effects of pharmacological treatment with HU on cognitive function, untreated and HU-treated trisomic Ts65Dn mice and euploid littermates were tested in the three-tier battery of water maze tasks. The serial water maze design measures differences in spatial learning and allows for a differential assessment of reference memory, episodic-like memory, and working memory (Jankowsky et al., 2005; Savonenko et al., 2005). In addition, the design of the probe trials with a collapsible platform increased the sensitivity of the tasks by detecting deficits or improvements in short- vs. long-term spatial memory as well as accessing memory acquisition at different stages of spatial learning (Andreasson et al., 2001). Each mouse performed a 4-day classic MWM task followed by 3 days of repeated reversals (RRWM) and a 4-day repeated reversal in a radial water maze (RAWM) (Figure 2). During daily probe trials, the hidden platform was lowered for a variable interval (30–45 s) and then raised to maintain the same response-reinforcement contingency as in the platform trials (Markowska et al., 1993; Micheau et al., 2004). Probe trials performed shortly after the training trials monitored short-term memory. Probe trials performed after an overnight delay and before the training trials monitored long-term memory. The mice were 11.8 ± 0.3 weeks old at the start of HU treatment and 23.3 ± 1.8 weeks old at the time of behavioral testing. Figure 1, 2 depict our study design and schedule of behavioral testing. Table 1 and Supplementary Tables S1–S3 summarize the statistical results for the water maze tasks.

### Classic Morris Water Maze (MWM)

**FIGURE 3:**
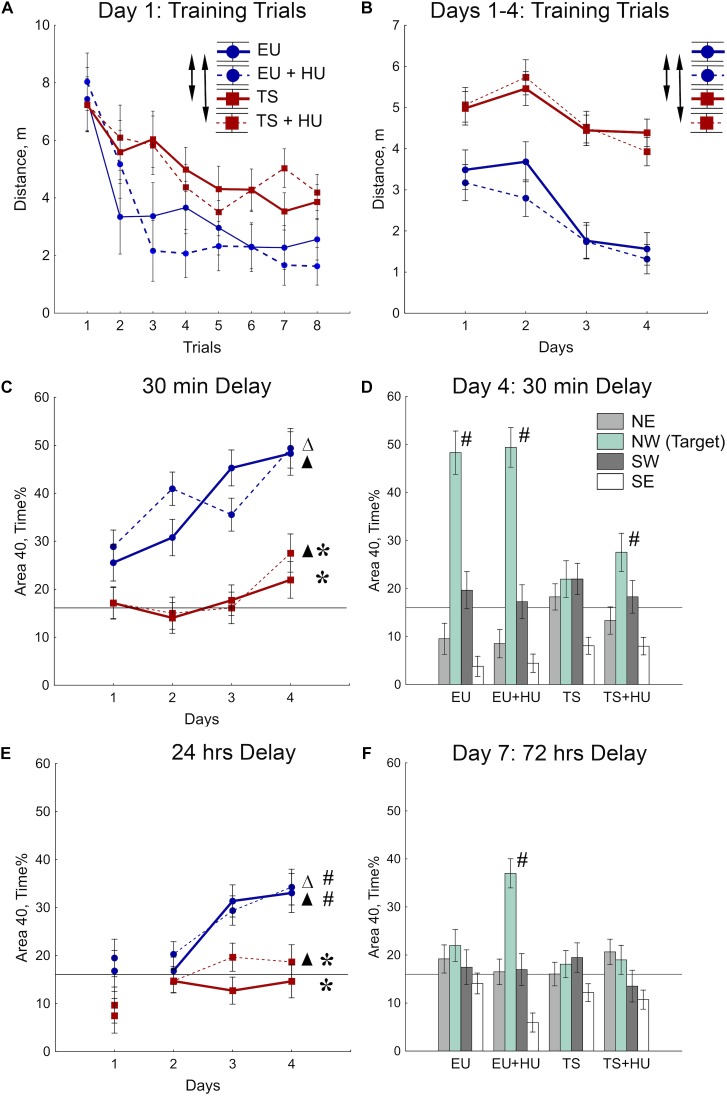
Trisomic Ts65Dn mice display deficits in spatial learning and memory in the MWM task. HU treatment marginally improved deficits in Ts65Dn mice and facilitated memory retention in control mice. **(A)** Day 1 learning dynamics. Group averages for distance traveled to the hidden platform during eight training trials on day 1. **(B)** Average distance traveled to the hidden platform across days. **(C)** Short-term probe trials conducted after a 30-min delay. Percent of time spent in the Area 40 platform area during the last probe trial of days 1–4. **(D)** Spatial preferences for different Areas 40 in the water maze. Percent of time spent in each Area 40 is shown for probe 2 (30-min delay) on Day 4 of the MWM task. **(E)** Long-term probe trials conducted after a 24-h delay. Percent of time spent in the NW Area 40 platform area during the first probe trial of days 1–4. **(F)** Spatial preferences for different Areas 40 after a 72-h delay. Arrows in **(A,B)** indicate significant differences from EU group (*p* < 0.005, LSD *post-hoc* test applied to significant main effect of Group in RM-ANOVA, see statistical results in Table 1). Asterisks in **(C,E)** indicate significant differences from EU group (*p* < 0.005, LSD *post-hoc* tests applied to a set of means at particular levels of Group × RM interaction in RM-ANOVA, Table 1). Triangles in **(C,E)** indicate significant differences between Day 1 and Day 4 (*p* < 0.05, LSD *post-hoc* test, Table 1). Pound signs in **(D–F)** indicate significant differences from the chance level for the NW Area 40 (*p* < 0.025, two-tailed *t*-test). Solid lines in **(C–F)** represent the chance level of performance during probe trials (16%). EU, euploid, *n* = 10. EU + HU, HU-treated euploid, *n* = 12. TS, Ts65Dn, *n* = 14. TS + HU, HU-treated Ts65Dn, *n* = 13. NE, northeast; NW, northwest; SE, southeast; SW, southwest.

The MWM was used to test spatial reference memory acquisition and retention (Morris, 1984; Vorhees and Williams, 2006, 2014). During the first day of MWM training, all four groups of mice improved their ability to locate the platform as indicated by a decrease in the distance swum to find the platform across training trials (Figure 3A and see Table 1 for statistical analysis). As expected, however, Ts65Dn mice traveled significantly greater distances to find the hidden platform as compared to euploid littermates indicating a spatial learning deficit. HU treatment did not significantly affect the performance of either genotype (Figure 3A). These differences between Ts65Dn and control mice observed during Day 1 of the MWM persisted through all 4 days of training in this reference memory task (Figure 3B). HU-treatment did not improve the Ts65Dn-related deficit (Figure 3B).

To test for spatial memory retention, short-delay (30 min) and long-delay (24 h) probe trials were introduced on each training day (Figure 2). In the short-delay probe trials, both control euploid groups (EU and EU+HU) increased their preference to the NW Area 40 platform area as training progressed (Figure 3C, Table 1, and Supplementary Table S1). In contrast, performance of Ts65Dn mice remained close to the chance level (16%) throughout the entire period of testing in the MWM task (Figure 3C,D). This was consistent with the deficit in acquisition of spatial memory observed in the TS group during the training trials (Figure 3A,B). The performance of HU-treated Ts65Dn mice (TS+HU) was also significantly worse compared to euploid littermates (Figure 3C). However, in contrast to the TS group, TS+HU mice significantly increased the time spent in the NW Area 40 platform area between Day 1 and Day 4 (Figure 3C). An assessment of the final accuracy of spatial reference memory (Day 4, Figure 3D) showed that the time spent by HU-treated Ts65Dn mice in the NW Area 40 platform area was significantly higher than the chance level (Supplementary Table S1). These data indicate that despite the dramatic deficits observed in the acquisition of spatial reference memory in Ts65Dn mice, HU-treatment significantly improved the accuracy of their memory when tested during the short-term delay.

In the probe trials with longer delays (24 or 72 h), none of the Ts65Dn groups showed performance better than the chance level (Figure 3E,F). Notably, the data from the 24-h delay probe trials indicated that the euploid mice successfully retained the location of the hidden platform (Figure 3E). However, only the HU-treated euploid mice showed significant recall of the platform location after a 72-h delay (Figure 3F). This finding implicates a nootropic effect of HU in control mice. Overall, HU-treatment most significantly improved the short-term memory of the HU-treated Ts65Dn mice (Day 4) and the long-term reference memory of the euploid mice.

### Repeated Reversal Water Maze (RRWM)

**FIGURE 4:**
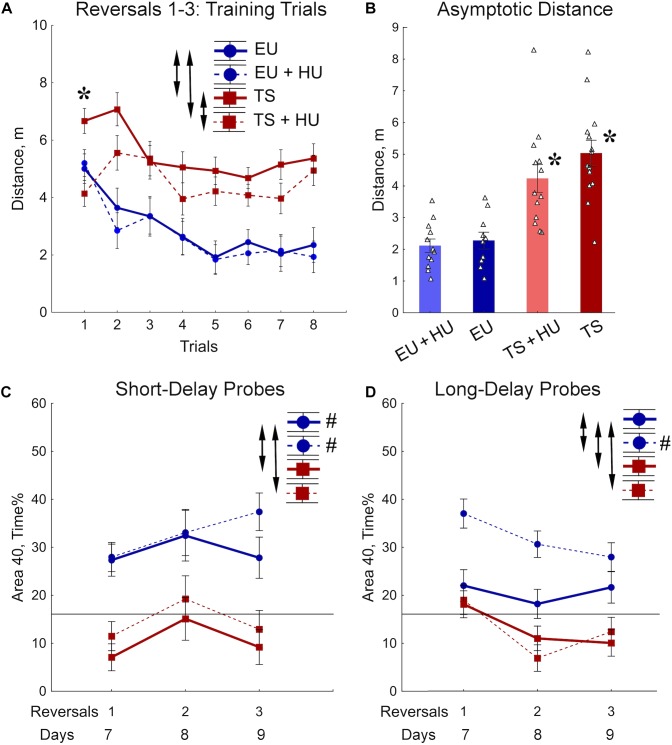
Trisomic Ts65Dn mice display deficits in acquisition of memory for new platform locations in the RRWM task. HU-treatment improved retention of new memories in euploid mice. **(A)** The group means of the distance traveled to the new platform locations averaged per trial across days. **(B)** Asymptotic level of performance shown as the distance averaged across trials 4–8. No effect of trial was observed during this period of training (Table 1). **(C)** Percent of time spent in the area 40 cm in diameter around a new platform location during the short-term probe trials with a 30-min delay. **(D)** Percent of time spent in the Area 40 surrounding the previous day’s platform as assessed in the long-term probe trials with a 72-h delay for reversal 1 and a 24-h delay for reversals 2 and 3. Arrows in panels **(A,C,D)** indicate significant differences from EU group (*p* < 0.05, LSD *post-hoc* test applied to significant main effect of Group in RM-ANOVA, see statistical results in Table 1). Asterisks in **(A,B)** – significant differences from EU group (*p* < 0.005, LSD *post hoc* test applied to main Group effect in one-way ANOVA, Table 1). Pound signs in **(C,D)** indicate significant differences between an average preference to new Area 40 platform locations and a chance level (*p* < 0.025, two-tailed *t*-test). Solid lines in **(C,D)** represent the chance level of performance during probe trials (16%). EU, euploid, *n* = 10. EU + HU, HU-treated euploid, *n* = 12. TS, Ts65Dn, *n* = 14. TS + HU, HU-treated Ts65Dn, *n* = 12.

During the RRWM the hidden platform was moved to a different pool quadrant each day for 3 days to assess the cognitive ability to learn a new platform location daily, cognitive plasticity (Figure 2A). The results of the 72-h MWM probe trial, the first probe trial of the RRWM task, demonstrated that the HU-treated euploid mice were the only group of mice to remember the location of the platform from the previous week’s testing. Thus, in contrast to other groups, HU-treated euploid mice had not only to learn the new platform location, but also inhibit visiting the previous platform. Despite this additional complexity, the performance of HU-treated euploid mice in the training trials was indistinguishable from the untreated euploid mice (Figure 4A,B). Both groups of Ts65Dn mice swam a significantly greater distance than control euploid mice to reach the new platform location (Figure 4A). Notably, HU-treatment resulted in mild but significant amelioration of the learning deficits in Ts65Dn mice (Figure 4A, Table 1, and Supplementary Table S2). These between-group differences were modulated across the training trials. In particular, the ameliorating effect of HU-treatment in Ts65Dn mice was the most pronounced in the first trial after the new platform location was introduced (Supplementary Table S2). Later in the training when the asymptotic level of performance was reached (Trials 4–8), the advantage of HU-treatment in Ts65Dn mice became marginal (Trials 4–8, Figure 4B).

Short- (30 min) and long-delay (24 h) probe trials revealed dramatic deficits in memory for the new platform location in both the untreated and HU-treated Ts65Dn mice (Figure 4C,D). These deficits were characterized by significantly poorer performance as compared to the euploid mice as well as by an inability to retain any spatial preference for a platform location higher than the chance level (16%; Figure 4C,D). These findings were consistent with the impairment in memory acquisition observed in trisomic mice during training trials (Figure 4B). In both groups of euploid mice, the retention of memory for the new platform location was above the chance level when tested shortly after the training trials (30 min short-delay; Figure 4C). However, after the 24 h long-delay, untreated euploid mice failed to show spatial preference. Notably, HU-treated euploid mice spent significantly more time in the Area 40 platform areas than untreated controls, and their performance was considerably above the chance level even after the 24 h long-delay (Figure 4D). Thus, the data from the RRWM task indicated that trisomic mice were unable to learn this complex episodic-like memory task. HU-treatment resulted only in a mild improvement of learning deficits in trisomic mice that did not result in better short- or long-term memory. In the euploid mice, HU-treatment significantly improved long-term memory of the platform locations without interfering with behavioral flexibility in acquisition of new spatial memories.

### Repeated Reversal Radial Arm Water Maze (RAWM)

**FIGURE 5:**
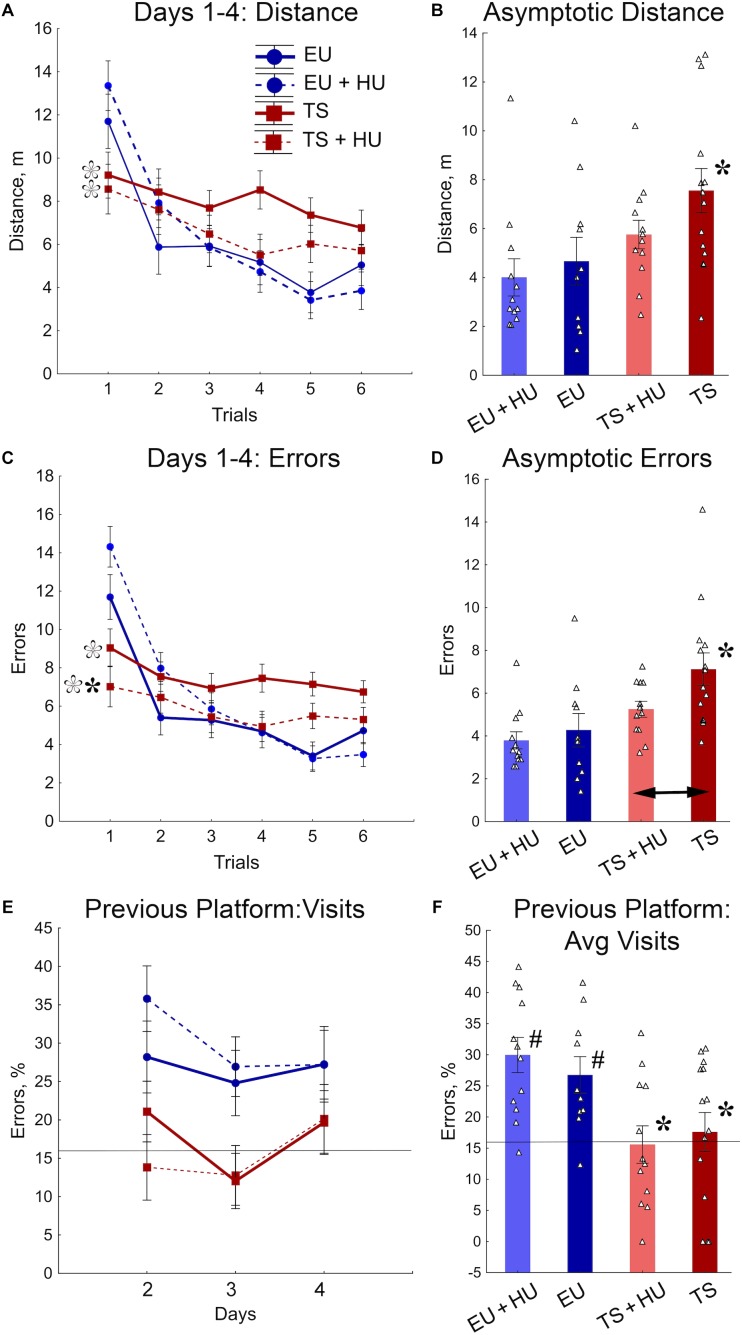
HU-treatment ameliorated deficits of trisomic Ts65Dn mice in acquisition of memory for new platform locations in the RAWM task. **(A)** Average distance to the platform per trial averaged across days. **(B)** Asymptotic performance assessed as average distance for trials 4–6. **(C)** Average number of errors per trial averaged across days. **(D)** Asymptotic performance assessed as average number of errors for trials 4–6. **(E)** Percent of errors due to visits to the previous day’s platform location during trial 1 (total number of entries to previous day’s platform arm/total number of arm entries × 100%). **(F)** Group means for percent of errors due to visits to the previous day’s platform location (as shown in **E**) averaged across days 1–3. Filled asterisks in **(B–D)** indicate significant differences from EU group (*p* < 0.02, LSD *post hoc* test applied to main Group effect in one-way ANOVA, Table 1). Empty asterisks indicate significant differences from EU + HU group (*p* < 0.01) and were added to panels **(A,C)** to explain significant effects of Group in trial 1 (one-way ANOVA, Table 1). Arrow in **(D)** indicates significant differences between TS and TS + HU groups (*p* < 0.05, LSD *post hoc* test applied to significant main effect of Group in ANOVA, Table 1). Pound signs in **(F)** indicate significant differences between levels of errors and a chance level (16.6%; *p* < 0.010, two-tailed *t*-test). Solid lines in **(E,F)** represent the chance level of performance (16.6%). *n* = 10. EU + HU, HU-treated euploid, *n* = 12. TS, Ts65Dn, *n* = 14. TS + HU, HU-treated Ts65Dn, *n* = 12.

All four groups of mice were tested in a repeated reversal RAWM. This task requires cognitive flexibility to learn a new platform location daily, similar to the RRWM. However, the RAWM protocol does not require procedural learning to stay around the platform location when the platform is absent as do the probe trials of the MWM and RRWM. In the RAWM task, the mice climb onto the platform as soon as they find it. Analyses of distance and number of errors before finding the platform revealed similar patterns of results for both variables (Figure 5A–D). The between-group differences were modulated across the training trials (Figure 5A,C, Table 1, and Supplementary Table S3). During the orientation trial, when a new location of the platform was first presented, the euploid mice showed longer distances and a higher number of errors than trisomic mice. During the asymptotic phase of training (trials 4–6), the between-group differences were characterized by significant deficits observed in untreated Ts65Dn mice as compared to untreated euploid controls. Notably, for both distance and the number of errors measured (Figure 5B,D), HU-treated Ts65Dn mice were not statistically distinguishable from euploid controls, indicating beneficial effects of the HU-treatment. Furthermore, HU-treated Ts65Dn mice made significantly fewer errors than did their untreated trisomic littermates (Figure 5D).

To determine if the longer distances and larger number of errors made by euploid mice during the first trials were due to their memory of the previous day’s platform location, the percentage of errors due to entry into the arm that contained the platform on the previous training day was analyzed (Figure 5E,F). Indeed, the number of visits to the previous day’s platform location was significantly greater for untreated and HU-treated euploid mice than Ts65Dn or HU-treated Ts65Dn mice. Moreover, in contrast to trisomic mice, the number of visits to the previous day’s platform was significantly higher than the chance level (16.6%; Figure 5F). None of the genotypes were significantly affected by HU-treatment in this measurement of performance (Figure 5E,F). Overall, the RAWM results suggest that HU-treatment in trisomic Ts65Dn mice improved their acquisition of spatial memory for a new platform location but did not improve the deficits in long-term memory retention.

## Discussion

Here we demonstrate that hydroxyurea (HU), an FDA-approved pharmacological agent known to induce the adaptive cellular stress response, can improve acquisition and retention of spatial memory in mice. One of the main novel findings is that HU treatment significantly improves long-term retention of reference and episodic-like memory in control mice, which is consistent with a robust nootropic effect. The beneficial effects of HU treatment in Ts65Dn mice are significant but limited. HU treatment only partially ameliorates some of the deficits exhibited by 6 month-old Ts65Dn mice in reference and episodic-like memories. These results also show the value of using the three-tier water maze design to more sensitively assess the cognitive deficits present in Ts65Dn mice and to better evaluate the therapeutic potential of pharmacological agents such as HU on learning and memory in both wildtype and genetically modified mice.

Since individuals with DS have more difficulty with working memory and episodic long-term memory than with implicit reference memory (Liogier d’Ardhuy et al., 2015), testing DS models and potential treatments using experimental designs that assess different memory systems has translational benefits. The three-tier water maze testing (Savonenko et al., 2005; Borchelt and Savonenko, 2008) begins with the classic MWM, a task that tests spatial reference memory and is the most widely used task to document spatial learning and memory deficits in Ts65Dn (Reeves et al., 1995; Moran et al., 2002; Seo and Isacson, 2005; Das et al., 2013; Dutka et al., 2015). Here we incorporated daily memory probe trials at the start and end of each MWM training session to monitor memory acquisition and retention after short and long delays. This schedule of probe trials revealed that despite significant improvements in the distance to reach the platform observed in Ts65Dn mice within a daily training session, there was no significant acquisition of spatial memory when tested at the end of daily training. These data indicate that the improvement observed in trisomic mice during training trials cannot solely be attributed to spatial learning but rather to engagement of non-spatial adaptive strategies. The robust deficit of Ts65Dn mice in the acquisition of spatial memory prevented incremental daily increases in reference memory as measured by the 4-day long MWM. However, HU-treatment produced mild but significant benefits in Ts65Dn by improving the short-term retention of spatial information as measured by a significant increase in time spent in the Area 40 platform area at the end of the MWM training compared to the chance level.

Less documented in Ts65Dn mice are deficits in spatial episodic-like and working memories. The repeated reversals task (RRWM) introduces an additional challenge of cognitive flexibility for learning a new platform location every day. A recent study, using a similar behavioral paradigm of reversal learning, indicated that diminished cognitive flexibility is the most robust cognitive impairment in Ts65Dn mice (Olmos-Serrano et al., 2016). Here, we show that at the stage of severe deficits in the acquisition of reference memory (discussed above) trisomic mice are unable to demonstrate behavior consistent with episodic-like memory. The consequences of this deficit were further detected in the radial water maze (RAWM) as an increase in the number of errors and distance swum to find the new locations of the hidden platform. Considering the severity of the deficits observed in Ts65Dn mice in both episodic-like memory tasks, the finding that HU treatment partially counteracted such severe cognitive impairment is remarkable. The beneficial effects of HU-treatment in the RRWM were limited to training trials, in particular the 1st trial after the mice were introduced to a new platform location (immediately after the 1st probe trial). In the RAWM, the beneficial effects of HU-treatment extended to more training trials which resulted in significant improvements of asymptotic performance levels. Some procedural differences between RRWM and RAWM tasks might support more sensitive detection of behavioral responses to HU-treatment in the latter task. The RAWM helps to circumvent thigmotaxic behavior that the Ts65Dn mice tend to show in a circular pool (Vorhees and Williams, 2006; Altafaj et al., 2013). One of the other main differences with RRWM is the lack of probe trials in the RAWM which bypasses the requirement of instrumental learning to stay around the platform when it is not readily available (Savonenko et al., 2005; Borchelt and Savonenko, 2008). Summarizing the observations from all three water maze tasks, the most common characteristic of cognitive deficits observed in trisomic mice was their inability to rapidly acquire new information for long-term storage, a feature akin to episodic-like memory. Despite the severity of cognitive impairments, HU-treatment mildly but significantly ameliorated these deficits in trisomic mice.

Multiple compounds have been tested for the ability to improve DS cognitive deficits in mouse models and human clinical trials (reviewed in Hart et al., 2017). Tested treatments have targeted neurogenesis (fluoxetine and lithium), *N*-methyl-D-aspartic acid (NMDA) receptor functioning (memantine), neurotrophin production, oxidative stress (vitamin E), and Alzheimer’s neuropathology (Lockrow et al., 2009; Bianchi et al., 2010; Netzer et al., 2010; Rueda et al., 2010, 2012; Begenisic et al., 2014). Additionally, a single postnatal dose of a sonic hedgehog pathway agonist, SAG, has been shown to normalize the morphology of the cerebellum as well as improving spatial learning and memory performance of Ts65Dn mice; a single injection of SAG on the day of birth normalizes learning in the MWM and its physiological correlate, long term potentiation, in adult mice (Das et al., 2013).

There are several possible reasons why the HU treatment in our study yielded only small improvements. Since this was the first study of this compound in a DS model, parameters for HU treatment have not been optimized. Changing the dosage and/or starting HU treatment at an earlier age may improve the efficacy of HU. Also, some brain abnormalities occur prenatally in DS, and therefore, neurodevelopmental defects are present at birth (Stagni et al., 2015). Most of neurogenesis occurs during the prenatal period. However, neurogenesis in the cerebellum does not stop until approximately 2.5 weeks after birth and is slowly ongoing in the dentate gyrus of the hippocampus. The effects of HU at the doses used here have not been studied *in utero*. Assuming prenatal HU treatment is safe, HU treatment may have the largest effect if started *in utero* or may have a larger effect on cerebellar and hippocampal development if administered neonatally (Stagni et al., 2015).

The pathways altered by HU may not have a significant effect on the structural and neurodevelopmental defects that are already present when the treatment started (∼3 month-old Ts65Dn mice). Some of the cognitive deficits experienced by individuals with DS are due to structural and developmental abnormalities. However, additional neuronal changes and decreases in cognition also occur during the lifetime of an individual with DS. In fact, approximately half of all individuals with DS will exhibit the neuropathology of Alzheimer’s-associated dementia by 60 years old (Head et al., 2012). This highlights the potential for therapeutic improvement of age-related cognitive decline in DS. In a mouse model, basal forebrain cholinergic (BFC) degeneration begins to occur at 6 months of age and continues throughout adulthood (Hunter et al., 2004; Kelley et al., 2014). Since BFC neurons are important for a variety of processes including learning/memory and attention (Hangya et al., 2015; Harrison et al., 2016), beginning HU treatment earlier and assessing the effects of HU treatment on a wider range of age-related cognitive deficits (1–12 months) may be informative for understanding a window of opportunity for HU treatment to be effective.

In our previous study, HU-treatment ameliorated the deficits in spatial reference memory in a model of Alzheimer’s disease, *APPswe/PS1dE9* mice (Brose et al., 2018). In contrast to that model, the efficacy of HU treatment in Ts65Dn mice was much more limited. The *APPswe/PS1dE9* mice, when tested in the MWM task with similar protocols (Savonenko et al., 2005; Borchelt and Savonenko, 2008; Liu et al., 2008), have less severe deficits in reference memory than that observed in trisomic mice. Although the absolute levels of memory measures in the *APPswe/PS1dE9* mice can be lower than in control mice starting from 6 to 8 months of age, their spatial preferences remain higher than chance level up to 18 months of age indicating robust reference memory. In contrast, the Ts65Dn mice tested at approximately 5–6 months of age in this study are not able to acquire any spatial preferences under similar protocols. The differences in the level of cognitive disability between the two disease models can be one of the reasons for diminished efficacy of HU treatment in the 5–6 month old Ts65Dn mice.

One of the unexpected findings of this study is the powerful nootropic effect of HU treatment observed in control mice. The HU-treated euploid mice were the only group of mice to accurately remember the platform location 72 h after the last day of the MWM training. The intact memory for the previous platform location increased the complexity of reversal learning for this group as they needed to re-write their memories to learn the new platform location. Nevertheless, the acquisition of memory for the new platform location in HU-treated euploid mice was as efficient as in their untreated littermates. Furthermore, HU-treated euploid mice successfully remembered the new reversed locations of the platform when tested 24 h later, a feature unreachable by untreated control mice. These nootropic effects of HU observed in the control mice were consistent with facilitation of memory retention in constant (reference memory) as well as time-variant conditions (episodic-like memory). Considering that episodic memory is particularly sensitive to aging, the data on HU nootropic effects suggest that this treatment may be beneficial to prevent such aging-related cognitive declines. Therefore, HU may be therapeutic for age-related dementia in DS and neurodegenerative diseases such as Alzheimer’s and Parkinson’s disease. Although we did not examine the molecular effects of HU in this study, we showed previously that HU treatment of cultured rat hippocampal neurons attenuated the loss of cell viability of neurotoxins that increase oxidative, metabolic, and excitotoxic stress, characteristics of neurodegenerative diseases. The neurotoxins tested included hydrogen peroxide, glutamate, rotenone, and amyloid beta peptide 1–42 (Brose et al., 2018). Furthermore, HU treatment of rat hippocampal neurons attenuated reductions of mitochondrial function induced by hydrogen peroxide treatment. Neurodegenerative disorders also exhibit defects in components of the adaptive cellular stress response pathways, pathways known to be upregulated by HU in human fibroblasts (Brose et al., 2012). Since neurodegenerative disorders tend to occur later in life, this leaves a large treatment window. The ability to diagnose these diseases at an early stage could be critical to treatment outcome.

## Conclusion

For future studies of therapeutic agents in Ts65Dn mice, we suggest the use of the three-tier water maze design because of its sensitivity to detect the effects of pharmacological agents on spatial learning and different types of memory including reference, episodic-like and working memories. Further studies in trisomic animal models should be completed to determine if a different dosage, different schedule or an earlier start of HU treatment may result in better efficacy. Additionally, the nootropic effects of HU on reference and episodic-like memory suggest the expansion of the therapeutic studies of HU to other neurological disorders with learning and memory deficits. The average lifespan of individuals with DS continues to increase. Identifying therapeutic treatments that improve the cognitive abilities of individuals with DS or delay cognitive decline offers a significant opportunity to positively affect the lives of DS individuals.

## Ethics Statement

This study was carried out in accordance with the recommendations of the NIH Guide for the Care and Use of Laboratory Animals and the Johns Hopkins University Institute of Animal Care and Use Committee. The protocol was approved by the Johns Hopkins University Institute of Animal Care and Use Committee.

## Author Contributions

RB, AS, KS, and RR contributed to conception and design of the study protocol. RB and BD performed the experimental protocols and collected the data. RB and AS analyzed and interpreted the data. RB and AS wrote the manuscript. All authors contributed significantly to preparation of the manuscript and have read and approved of the final manuscript.

## Conflict of Interest Statement

The authors declare that the research was conducted in the absence of any commercial or financial relationships that could be construed as a potential conflict of interest.
