# Supplementary material for: Hydroxyurea Improves Spatial Memory and Cognitive Plasticity in Mice and Has a Mild Effect on These Parameters in a Down Syndrome Mouse Model
Source: Front Aging Neurosci. 2019 May 14;11:96. doi: 10.3389/fnagi.2019.00096 (PMC6527804; doi:10.3389/fnagi.2019.00096)
Supplement: Supplementary file 1 [file Data_Sheet_1.PDF]

Supplemental Figure 1. Average age for each group of mice at the start of behavioral testing. Number of mice per group is indicated at the bottom of each column.

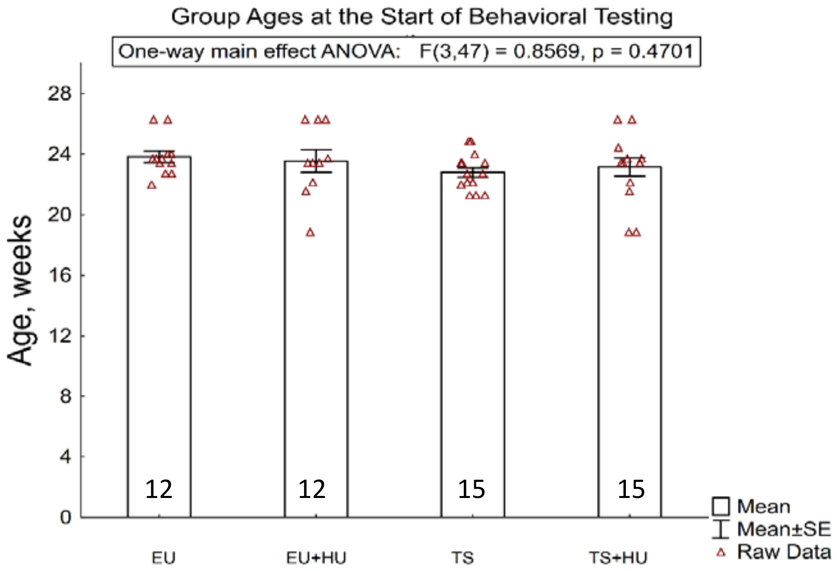

Supplemental Table 1: Details of Stat Results for the MWM task

Classical Morris Water maze (MWM)

**Figure**		**ANOVA**			**LSD post hoc**	
	Effect	dF	F	p	Sets of means	p level
					For GROUPS:	
3A	GROUP	3;45	5.20	0.003622	EU vs EU+HU	0.629446
	TRIAL	7;315	13.00	0.000000	EU vs TS	0.003153
	INTERACTION	21;315	0.95	0.523800	EU vs TS+HU	0.004061
					TS vs TS+HU	0.881730
3B					*For GROUPS:*	
	GROUP	3;45	32.16	0.000000	EU vs EU+HU	0.319690
	DAY	3;135	17.23	0.000000	EU vs TS	0.000000
	INTERACTION	9;135	0.81	0.604901	EU vs TS+HU	0.000000
					TS vs TS+HU	0.988493
3C					*Day1 vs Day4*	
	GROUP	3;45	22.3975	0.000000	EU	0.000008
	PROBE	3;135	16.4156	0.000000	EU+HU	0.000006
	INTERACTION	9;135	2.6941	0.006505	TS	0.238516
					TS+HU	0.015658
					Day4: For GROUPS:	
					EU vs EU+HU	0.839481
					EU vs TS	0.000138
					EU vs TS+HU	0.000001
					TS vs TS+HU	0.252365
3D					*NW: For GROUPS:*	
	GROUP	3;45	4.501	0.007609	EU vs EU+HU	0.819680
	QUADRANT	3;135	54.003	0.000000	EU vs TS	0.000020
	INTERACTION	9;135	5.677	0.000001	EU vs TS+HU	0.000000
					TS vs TS+HU	0.199562
					t-test: NW vs Chance level	
					EU	0.00010
					EU+HU	0.00001
					TS	0.18040
					TS+HU	0.02150
3E					*Day1 vs Day4*	
	GROUP	3;45	12.0466	0.000006	EU	0.001246
	PROBE	3;135	12.1964	0.000000	EU+HU	0.001305
	INTERACTION	9;135	1.3374	0.223375	TS	0.087572
					TS+HU	0.039604
					Day4: For GROUPS	
					EU vs EU+HU	0.807152
					EU vs TS	0.003836
					EU vs TS+HU	0.000191
					TS vs TS+HU	0.370864
3F					*NW: For GROUPS:*	
	GROUP	3;44	4.281	0.009780	EU vs EU+HU	0.000392
	QUADRANT	3;132	12.044	0.000001	EU vs TS	0.470554
	INTERACTION	9;132	3.215	0.001485	EU vs TS+HU	0.333450
					TS vs TS+HU	0.898716
					t-test: NW vs Chance level	
					EU vs EU+HU	0.13390
					EU vs TS	0.00001
					EU vs TS+HU	0.36500
					TS vs TS+HU	0.37360

Supplemental Table 2: Details of Stat Results for the RRWM task

Repeated Reversal Water Maze (RRWM)

**Figure**		**ANOVA**			**LSD post hoc**	
	Effect	dF	F	p	Sets of means	p level
4A					*For GROUPS:*	
	GROUP	3;45	14.4017	0.000001	EU vs EU+HU	0.741491
	TRIAL	7;315	12.0475	0.000000	EU vs TS	0.000007
	INTERACTI(	21;315	2.0761	0.004155	EU vs TS+HU	0.003505
					TS vs TS+HU	0.043552
	Orientation Trial (Trial 1)				*For GROUPS:*	
	GROUP	3;45	14.4017	0.000001	EU vs EU+HU	0.780675
					EU vs TS	0.017348
					EU vs TS+HU	0.213279
					TS vs TS+HU	0.000210
4B	Asymptotic Trials (Trials 4-8)				*For GROUPS:*	
	GROUP	3;45	16.1515	0.000000	EU vs EU+HU	0.764512
	TRIAL	4;180	0.7172	0.581174	EU vs TS	0.000004
	INTERACTI(	12;180	0.5351	0.889802	EU vs TS+HU	0.000645
					TS vs TS+HU	0.107543
4C					*For GROUPS:*	
	GROUP	3;44	17.3474	0.000000	EU vs EU+HU	0.363474
	DAY	2;88	3.1322	0.048520	EU vs TS	0.000013
	INTERACTI(	6;88	0.5574	0.763025	EU vs TS+HU	0.000561
					TS vs TS+HU	0.264585
					t-test: AVG vs Chance level	
					EU	0.00100
					EU+HU	0.00001
					TS	0.03590
					TS+HU	0.57960
4D					*For GROUPS:*	
	GROUP	3;44	20.8431	0.000000	EU vs EU+HU	0.000422
	DAY	2;88	8.7209	0.000350	EU vs TS	0.011197
	INTERACTI(	6;88	0.9449	0.467380	EU vs TS+HU	0.010961
					TS vs TS+HU	0.917455
					t-test: AVG vs Chance level	
					EU	0.0533
					EU+HU	0.0000
					TS	0.1161
					TS+HU	0.1181

Supplemental Table 3: Details of Stat Results for the RAWM task

Repeated Reversal Radial Water Maze (RAWM)

**Figure**		**ANOVA**			**LSD post hoc**	
	Effect	dF	F	p	Sets of means	p level
5A						
	GROUP	3;44	1.0308	0.388264		
	TRIAL	5;220	41.6306	0.000000		
	INTERACTI(	15;220	5.6905	0.000000		
	Orientation Trial (Trial 1)				*For GROUPS:*	
	GROUP	3;44	14.4017	0.000001	EU vs EU+HU	0.338220
					EU vs TS	0.138518
					EU vs TS+HU	0.072877
					TS vs TS+HU	0.682210
5B	Asymptotic Trials (Trials 4-6)				*For GROUPS:*	
	GROUP	3;44	3.8301	0.016021	EU vs EU+HU	0.588987
	TRIAL	2;88	3.8412	0.025155	EU vs TS	0.018295
	INTERACTI(	6;88	1.8865	0.091972	EU vs TS+HU	0.376493
					TS vs TS+HU	0.115193
5C						
	GROUP	3;44	1.7808	0.164728		
	TRIAL	5;220	51.0061	0.000000		
	INTERACTI(	15;220	8.8872	0.000000		
	Orientation Trial (Trial 1)				*For GROUPS:*	
	GROUP	3;44	8.9073	0.000100	EU vs EU+HU	0.104019
					EU vs TS	0.089198
					EU vs TS+HU	0.004966
					TS vs TS+HU	0.171424
5D	Asymptotic Trials (Trials 4-6)				*For GROUPS:*	
	GROUP	3;44	6.2285	0.001280	EU vs EU+HU	0.595875
	TRIAL	2;88	2.1696	0.120300	EU vs TS	0.002276
	INTERACTI(	6;88	1.7746	0.113515	EU vs TS+HU	0.289511
					TS vs TS+HU	0.029941
5E-F					*For GROUPS:*	
	GROUP	3;44	5.3361	0.003187	EU vs EU+HU	0.476113
	DAY	2;88	2.6196	0.078506	EU vs TS	0.040338
	INTERACTI(	6;88	0.8547	0.531529	EU vs TS+HU	0.016540
					TS vs TS+HU	0.627389
					t-test: AVG vs Chance level	
					EU	0.01030
					EU+HU	0.00070
					TS	0.71940
					TS+HU	0.73130
